# Two Distinct Conformations in Bet v 2 Determine Its Proteolytic Resistance to Cathepsin S

**DOI:** 10.3390/ijms18102156

**Published:** 2017-10-16

**Authors:** Wai Tuck Soh, Peter Briza, Elfriede Dall, Claudia Asam, Mario Schubert, Sara Huber, Lorenz Aglas, Barbara Bohle, Fatima Ferreira, Hans Brandstetter

**Affiliations:** 1 Department of Molecular Biology, University of Salzburg, Salzburg 5020, Austria; waituck.soh@sbg.ac.at (W.T.S.); Peter.Briza@sbg.ac.at (P.B.); Elfriede.dall@sbg.ac.at (E.D.); claudia.asam@sbg.ac.at (C.A.); mario.schubert@sbg.ac.at (M.S.); Sara.Huber@stud.sbg.ac.at (S.H.); lorenz.aglas@sbg.ac.at (L.A.); Fatima.Ferreira@sbg.ac.at (F.F.); 2 Department of Pathophysiology, Medical University of Vienna, Vienna 1090, Austria; barbara.bohle@meduniwien.ac.at

**Keywords:** post-translational modification, allergen, endolysosomal proteases, panallergen

## Abstract

Birch pollen allergy affects more than 20% of the European allergic population. On a molecular level, birch pollen allergy can be linked to the two dominant allergens Bet v 1 and Bet v 2. Bet v 2 belongs to the profilin family, which is abundant in the plant kingdom. Importantly, the homologous plant profilins have a conserved cysteine motif with a currently unknown functional relevance. In particular, it is unknown whether the motif is relevant for disulfide formation and to what extent it would affect the profilins’ structural, functional and immunological properties. Here we present crystal structures of Bet v 2 in the reduced and the oxidized state, i.e., without and with a disulfide bridge. Despite overall structural similarity, the two structures distinctly differ at their termini which are stabilized to each other in the oxidized, i.e., disulfide-linked state. These structural differences translate into differences in their proteolytic resistance. Whereas the oxidized Bet v 2 is rather resistant towards the endolysosomal protease cathepsin S, it is rapidly degraded in the reduced form. By contrast, both Bet v 2 forms exhibit similar immunological properties as evidenced by their binding to IgE antibodies from birch pollen allergic patients and by their ability to trigger histamine release in a humanized rat basophilic leukemia cells (RBL) assay, independent of the presence or absence of the disulfide bridge. Taken together our findings suggest that the oxidized Bet v 2 conformation should be the relevant species, with a much longer retention time to trigger immune responses.

## 1. Introduction

Birch pollen allergy affects over a hundred million people worldwide. In Europe, approximately 20% of allergic patients are allergic to birch pollen [1]. Bet v 1 and Bet v 2 are the two clinically most relevant allergens in birch pollen allergy, recognized by more than 95% and up to 40% of the birch pollen allergic patients, respectively, depending on geographical regions [2,3]. Bet v 2 is a plant profilin panallergen, i.e., it is ubiquitously distributed throughout nature and shares a highly conserved amino acid sequence of up to 75% even between distantly-related organisms [4]. This sequence conservation translates into a high similarity of the structural fold and function within other members of the profilin family [5]. Conversely, it also results in a broad IgE cross-reactivity of profilin allergic patients to other inhalant and nutritive allergen sources [6]. However, their sensitizing capabilities remain unclear.

Physiologically, profilins are known to play a role in cell elongation, cell shape maintenance, the growth of pollen tubes and root hairs in plants [7]. Additionally to its ability to bind actin, profilins also interact with poly-l-proline and phosphoinositides [4]. Due to the high similarity among profilin family members, properties obtained from a particular profilin are often transferred by similarity to other members. As an example, detailed analyses of the profilin sequences and structures suggested the presence of an intra-molecular disulfide bridge in several members including Bet v 2 [8]. A recent study showed that rubber tree profilin, Hev b 8, was able to homodimerize via an inter-molecular disulfide bridge [9]. Another study also observed an intra-disulfide bond between Cys95 and Cys117 in the three cysteines containing Art v 4 crystal structure, however, it is not observed in all the molecules [10]. It needs to be pointed out that the cysteine 95 is not conserved in profilin. By contrast, Bet v 2 contains only two conserved cysteines (Cys13 and Cys117), which were expected to form a disulfide bridge. The available Bet v 2 crystal structure (pdb entry 1cqa) did not confirm this conclusion, although the structure was obtained under reducing conditions. The existence of such post-translational modifications could influence the antigen stability toward endo-lysosomal proteases, which ultimately may impact the allergenicity of Bet v 2.

In this study, we present crystal structures of Bet v 2 in the reduced (without disulfide bridge) and the oxidized (with disulfide bridge) states at a resolution of 2.0 and 1.7 Å respectively. Furthermore, we examined the relevance of the existence of the intra-molecular disulfide bond with respect to structural, biochemical and immunological characteristics. Importantly, the observed differences are independent of the presence of reducing agents, reflecting the conformational differences of Bet v 2 in the presence and the absence of the disulfide bridge.

## 2. Results

### 2.1. Isolation and Cloning of Bet v 2

There are two Bet v 2 sequences reported in the protein database (www.uniprot.org) designated as profilin-I (P25816) and profilin-II (A4K9Z8). Detailed analysis of the birch pollen proteome confirmed the presence of a Bet v 2 isoform which corresponded to sequence entry A4K9Z8 [11]. Due to such inconsistency, we isolated the Bet v 2 gene sequence from birch pollen total RNA and the deduced amino acid sequence corresponded to the sequence A4K9Z8 (Figure S1a), i.e., profilin II.

### 2.2. Recombinantly-Produced Bet v 2 Exists in Two Conformations

**Figure 1:**
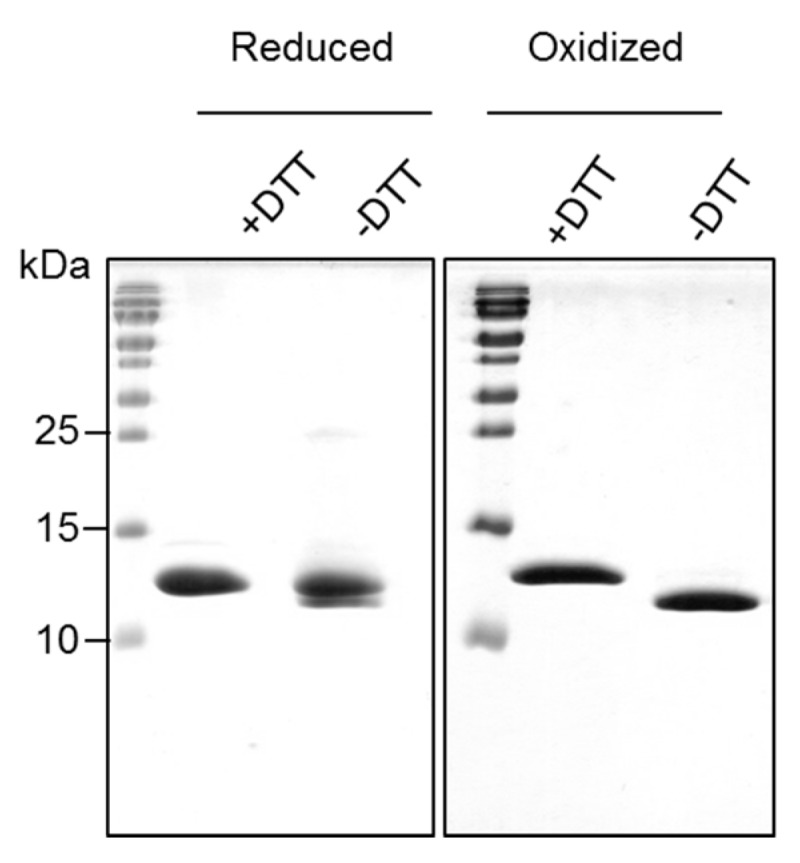
Migration of purified reduced (no disulfide) and oxidized (with disulfide) forms of recombinant Bet v 2 on 18% SDS-PAGE under non-reducing (−DTT) and reducing (+DTT) conditions. The “reduced” sample contains two species with apparently different molecular weights. The corresponding differences in compactness suggest a partial disulfide bridge formation. Proteins were visualized by Coomassie Blue staining. Please note the faint dimer band at 28 kDa which is present in the “reduced” Bet v 2 sample in the absence of DTT.

The birch pollen-derived Bet v 2 was successfully expressed as a soluble protein in *E. coli* and was found to bind poly-l-proline resin, confirming its correct folding. Part of Bet v 2 eluted from the poly-l-proline resin under ionic washing conditions (150 mM NaCl), whereas the majority of Bet v 2 bound tightly and was only eluted upon denaturing the protein with 6 M urea. This observation suggested that Bet v 2 existed in two conformations with low and high proline binding affinity. The identities of both fractions were confirmed by mass spectrometry as full-length proteins (Figure S1b). The two fractions migrated identically on a reducing SDS-PAGE at a size consistent with its mass (14.3 kDa). By contrast, on non-reducing SDS-PAGE one fraction appeared more compact and moved faster than the reduced lane, suggesting that the two conformers differ by a disulfide-bridge formation between the two conserved cysteines, Cys13 and Cys117 (Figure 1 and Figure S1a). We refer to the two conformations as the reduced (i.e., no disulfide) and the oxidized (disulfide-bridged) form. Indeed, mass spectrometry analyses further confirmed the presence of an intra-molecular disulfide bond in the oxidized form, whereas the majority (89%) of the reduced form did not contain a disulfide bond. However, there was approximately 11% of contamination with the oxidized form (Table 1). The yields of the reduced and oxidized forms were approximately 5 and 10 mg/L of culture, respectively.

### 2.3. Crystal Structure Analysis of Bet v 2 in the Presence (Oxidized) and Absence (Reduced) of A Disulfide Bridge

**Figure 2:**
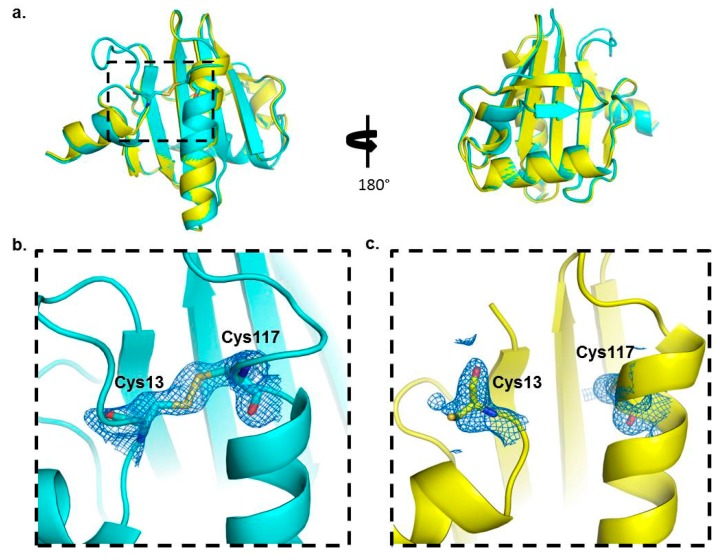
Crystal structures of Bet v 2. (**a**) Superimposition of the overall structure of oxidized and reduced forms. Bet v 2 oxidized and reduced forms are presented in cyan and yellow, respectively. Cys13 and Cys117 are shown in a stick representation; (**b**) Close-up view of the terminal region of oxidized Bet v 2 with disulfide bridge formed and (**c**) Close-up view of the terminal region of reduced Bet v 2 with cysteines in an open conformation. The density is displayed by 2Fo-Fc Map (*σ* = 1).

We further questioned what impact the disulfide might have on the structure and function of Bet v 2, and therefore set out to crystallize the reduced and oxidized Bet v 2. The reduced and oxidized structures were determined at a high resolution of 2.0 Å and 1.7 Å, respectively (Table 2). Both structures share a central six-stranded antiparallel β-sheet with two N- and the C-terminal α-helices flanking the sheet at one side and a third helix flanking the sheet on the opposite side (Figure 2a). In the oxidized form, the N-terminal segment is disulfide linked to the C-terminal helix, which adopts a strained conformation enforced by the disulfide bond. By contrast, in the reduced form the helix is more relaxed and extends beyond Cys117, which is rotated by approximately 30° between the oxidized and reduced structures (Figure 2b,c). Moreover, the N-terminal segment harboring Cys13 was moved away from the helix for approximately 13 Å and is partially disordered. In fact, Cys13 is visible in the electron density only in one of the two molecules within the asymmetric unit, and Asp14 to Gln21 is disordered in both molecules of the asymmetric unit.

To substantiate that the rearrangement upon disulfide formation or reduction is localized to the terminal regions, we calculated Cα-rms deviations for the central β-sheet (residue 22–110) and the terminal segments (N-terminal residue 2–12; C-terminal residue 111–133) separately, with values of 0.304 and 1.143 Å, respectively. The higher degree of disorder in the reduced form, lacking a disulfide bridge, is also reflected in the overall Cα-rmsd of 0.744 Å between the two molecules in the asymmetric unit of the reduced Bet v 2 crystal.

### 2.4. Structural Studies in Solution

To test whether the differences as deduced from the crystal structure analysis are significant also in solution, we employed several spectroscopic methods. Along this line, we collected one-dimensional ^1^H-NMR spectra of the oxidized (disulfide-bridged) and the reduced (no disulfide) Bet v 2 to compare the fingerprint of the upfield-shifted methyl groups that are sensitive to the structure and dynamics mainly of the core of these proteins (Figure S2). Indeed, their spectra revealed marked differences, consistent with the order–disorder transition, as deduced from the crystal structure analysis. The differences in the spectra are suggested to be related to the disulfide bridge, because upon the DTT-reduction of the oxidized Bet v 2 sample the ^1^H-NMR spectrum was widely identical to the reduced spectrum with the exception of peaks four and five, which showed small deviations. We suggest that these differences might result from the presence of the 11% disulfide-containing variant in the “reduced” protein (Table 1). However, both the oxidized and reduced forms have an exactly identical spectrum in the presence of DTT.

To test the secondary structure content, we pursued CD and FTIR spectroscopy. Both methods revealed a slight increase of helical content in the reduced Bet v 2 form as compared to the oxidized form (Figure S3). This increase reflects the higher ordering in the C-terminal helix, as revealed by the crystal structure analysis.

### 2.5. Functional Relevance of the Disulfide Bridge

We questioned to which extent the observed disorder–order transition upon the disulfide-bridge formation in Bet v 2 would translate into its allergenic properties. Thermal stability and proteolytic resistance have been shown to critically influence the immunological properties of allergens, in particular with respect to its processing and MHCII presentation [12].

We tested the global stability of Bet v 2 by the thermal unfolding of both redox forms using the thermal shift assay. Interestingly, both redox forms exhibited a virtually identical melting temperature of around 53 °C (Figure S4), reflecting that the global stability of Bet v 2 is hardly affected by the disulfide bridge.

**Figure 3:**
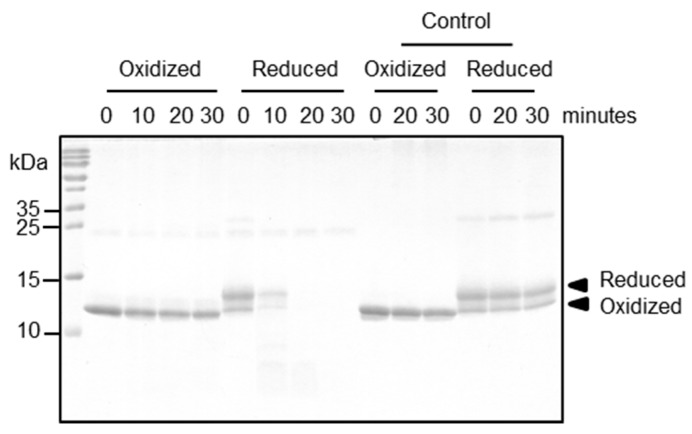
Proteolytic susceptibility of Bet v 2 towards cathepsin S. A time series digestion assay was performed at pH 5.5, 37 °C up to 30 min with cathepsin S (24 kDa) to Bet v 2 molar ratio of 1:20. Digestion profiles were visualized on SDS-PAGE under non-reducing condition and Coomassie Blue staining. As a control, Bet v 2 was monitored in the absence of a protease.

More relevant to antigen processing and presentation, we studied the proteolytic resistance of both Bet v 2 redox forms in the presence of the endo/lysosomal proteases cathepsin S and legumain. Legumain is a cysteine protease cleaving after Asn and at low pH also after Asp [13], whereas cathepsin S has a broader specificity with high relevance in antigen processing [14]. We found that legumain was slowly cleaving and degrading both Bet v 2 redox forms at a comparable rate (Figure S4b). Importantly, cathepsin S showed a strong preference for the reduced Bet v 2 isoform, which was completely degraded after 10 min. By contrast, the oxidized Bet v 2 isoform was resistant to cathepsin S degradation for more than 30 min (Figure 3), consistent with analogous reports on the proteolytic resistance of Bet v 2 to α-chymase [15]. It is noticeable that a faint dimer band (~28 kDa) was present in the reduced form and was also degraded by cathepsin S. Consequently, the intra-disulfide bridge protects Bet v 2 against cleavage by the antigen processing protease cathepsin S.

### 2.6. Immunological Properties of Bet v 2

**Figure 4:**
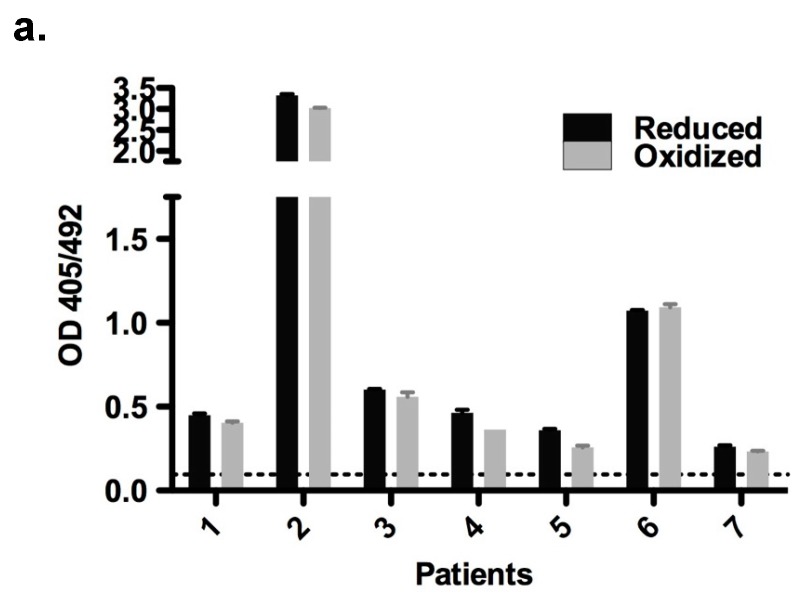

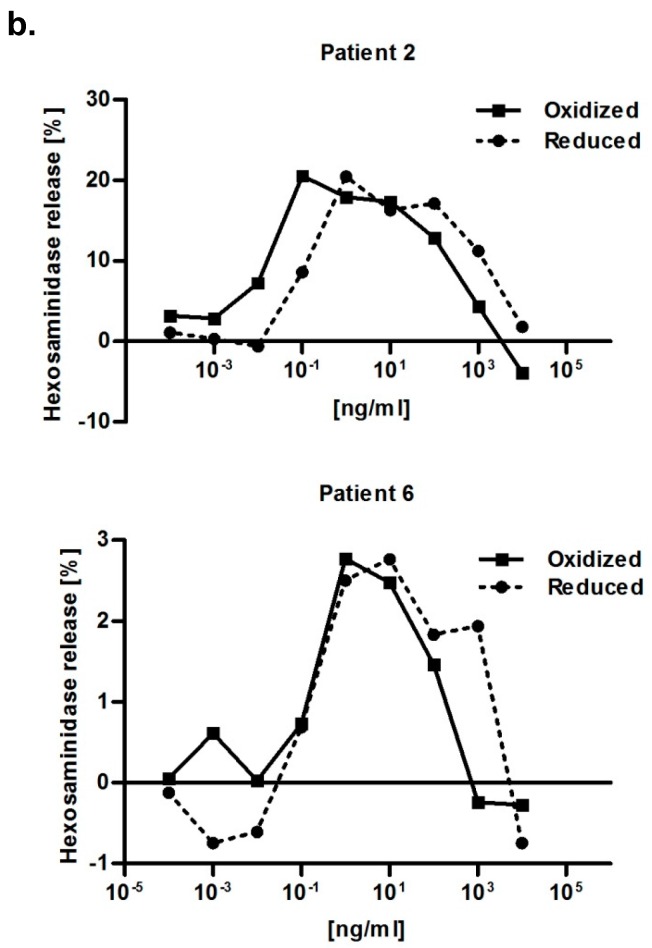
Immunological reactivity of Bet v 2. (**a**) ELISA assay of birch pollen allergic patients reactive to Bet v 2. The dashed line represents the limit of detection calculated from blank control with three times standard deviation; (**b**) Mediator release assay of the two birch pollen allergic patients with highest IgE binding as observed in (**a**); Patient two and six correspond to the numbers in the ELISA in panel (**a**). A non-allergic individual showed no release of hexosaminidase (data not shown). The shown hexosaminidase release was background corrected.

Given the drastically different recognition of the Bet v 2 redox forms by cathepsin S, we further tested whether both redox forms are recognized by IgE antibodies of birch pollen allergic patients. Using ELISA, we found that there was no statistically significant difference in the binding affinity (*p* = 0.069), suggesting that both Bet v 2 redox forms are relevant in the allergic patients (Figure 4a). To further evaluate the ability to trigger basophil degranulation, we carried out a mediator release assay using a rat basophil leukemia (RBL) cell line expressing the human Fcε type-1 IgE receptor. Sera from patient two and patient six were used for the RBL release assay, because they contained high IgE levels towards Bet v 2, as shown by the ELISA. Bet v 2 could induce histamine release in the RBL assay using these patient sera. The release was significantly higher for the serum of patient two than for serum of patient six, consistent with a significantly higher IgE titer in the ELISA (Figure 4a,b). These results again confirm the relevance of both redox forms for the allergenic properties of Bet v 2. Given the significantly higher proteolytic resistance of oxidized Bet v 2, we suggest that this form is the sensitizing allergen.

## 3. Discussion

A broad variety of post-translational modifications are known to regulate the stability and function of proteins, including glycosylation, phosphorylation, and disulfide formation [16]. While the endo-lysosome is equipped with an array of hydrolases to remove many of these modifications, the removal is often incomplete. Indeed, several of these modifications persist during antigen processing, thereby influencing the recognition by and stimulation of immune cells [16]. This is particularly true for disulfide bond formation, which has been previously shown to have the capacity to modulate T-cell recognition [17,18].

The correct post-translational modification is dependent on the host organism, which explains why the authentic modifications are sometimes not obtained in recombinantly-produced proteins, and are therefore often under-appreciated. As an example, heterologous expression in *E. coli* cells will typically produce proteins without disulfides formed, because the preferred cytosolic expression has a reducing environment. However, bioinformatic analysis and strict sequence conservation suggested that Bet v 2 should contain a disulfide bridge (Figure S1a) [8]. By contrast, the reported crystal structure of Bet v 2 lacked a disulfide bridge (PDB entry 1cqa), leaving open the question of whether a disulfide bridge might have formed under non-reducing protein production and crystallization protocols, i.e., whether Bet v 2 has the intrinsic property to form a disulfide bond.

These expectations were further reinforced by the strict conservation of the cysteine residues of the proposed disulfide in related plant profilins (Figure S1a). Furthermore, Thr113 was suggested to be phosphorylated in Bet v 2 by sequence similarity, albeit not strictly conserved, and also not supported by a birch pollen proteome analysis [11]. Therefore, additional experimental data were critical to evaluate the possible Cys13-Cys117 disulfide bond and its possible relevance.

**Figure 5:**
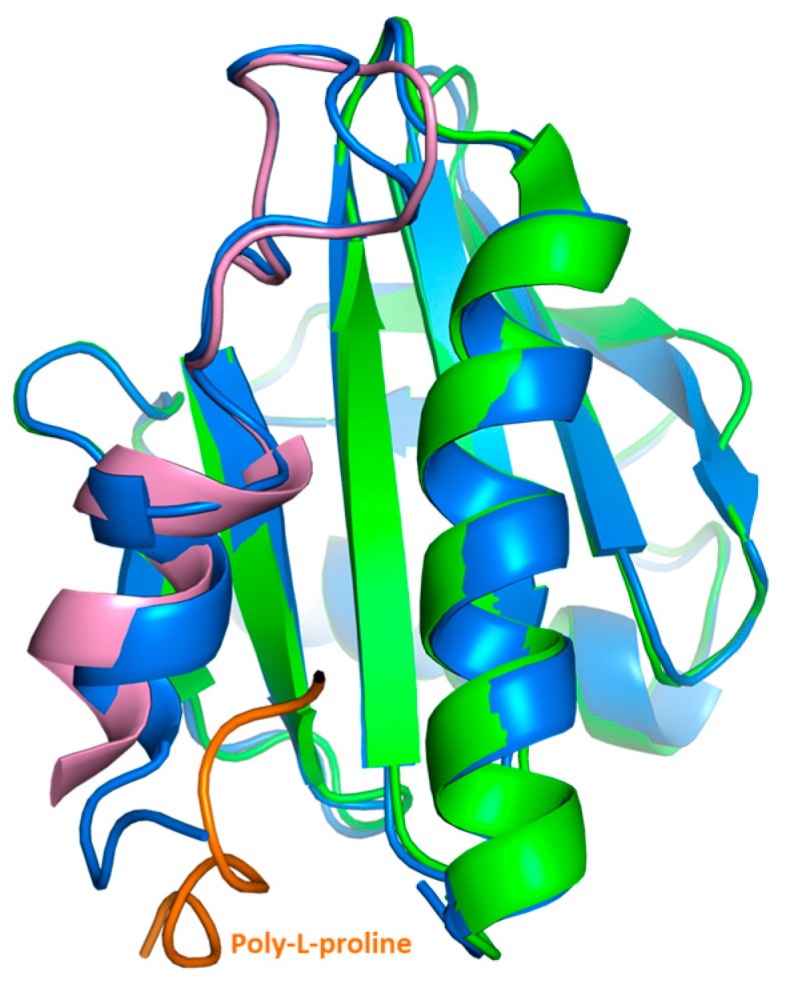
Superimposition of crystal structures of ragweed profilin (Amb a 8) alone (apo) and in complex with poly-(L-proline)_10_ ligand. The apo (pdb 5EM1) and complex (5EVE) structures of Amb a 8 are presented in blue and green, respectively. Poly-(L-proline) peptide is highlighted in orange, while the N-terminal segment, which undergoes an induced fit upon binding, was highlighted in pink.

Our crystal structure analysis revealed that Bet v 2 can indeed adopt two distinct conformations, representing the reduced and the oxidized, i.e., disulfide-bridged form. While the overall structure was conserved between these two forms, the crystal structures revealed marked differences near the N- and C-terminal segments, which are linked by the disulfide bridge Cys13-Cys117 (Figure 2). These differences were also manifested in solution by a significantly higher binding affinity of the oxidized (disulfide-bonded) than the reduced form towards poly-l-proline resin, as evidenced by a preferential elution of the reduced Bet v 2 form. Recently, a crystal structure of the complex of latex profilin with poly-l-proline has revealed the direct involvement of the terminal helices in ligand binding [10]. The poly-l-proline binding sites of Bet v 2 map onto the immediate neighborhood of the Cys13-Cys117 disulfide bond, providing a direct link between the differences in proline binding with the differences in the crystal structures of both Bet v 2 forms (Figure 5). Hence, the apparent higher binding ability to poly-l-proline of the oxidized Bet v 2 can be explained by the conformational selection principle: the likelihood for the ligand to find the receptor in a conformation suitable for binding is increased if the conformational space of the receptor is restricted by a disulfide bridge.

Both redox forms exhibited comparable allergenic reactivity with respect to IgE binding and histamine release (Figure 4). Further immunological conclusions would require a broader data base, i.e., a larger patient cohort—which is intrinsically difficult when working with a minor allergen. Importantly, however, the two redox forms differed strongly with respect to their proteolytic resistance, which can have major impacts on the immunologic properties of Bet v 2 for several reasons: firstly, given the long storage times of birch pollen proteins before the contact with a patient, the preferential degradation of the reduced Bet v 2 will accumulate the oxidized Bet v 2 form, resulting in a preferential exposure of patients with the more stable oxidized Bet v 2 form. Secondly, the process of allergic sensitization is intimately linked to the proteolytic processing of the allergen preceding the MHCII presentation. In this work, we showed that cathepsin S degrades the reduced form of Bet v 2 rapidly, whereas the processing of the oxidized form is sufficiently retarded. Cathepsin S has been shown to be responsible in the generation of most Bet v 1 peptide repertoire [19]. In previous studies we showed that Bet v 1.0101 was more resistant towards cathepsin S as compared to Bet v 1.0104. This resistance in Bet v 1.0101 resulted in fewer peptides available for MHCII presentation and correlated with a stronger Th2 polarization as compared to Bet v 1.0104 [20]. These results were supported and refined by in vivo studies, demonstrating that an increase in the fold stability of Bet v 1.0101 enhances its allergenicity and immunogenicity [12]. These studies support the notion that resistance to endolysosomal protease is an intrinsic feature of a sensitizing allergen. Thirdly, the disulfide-mediated stabilization of the N-terminal segment is likely to prevent or attenuate Bet v 2 cleavages at Tyr6-Val7 and Trp35-Ala36 by α-chymase, which gets released from mast cells. These cleavages were found to affect and destruct IgE epitopes [15,21]. These findings together suggest that the Cys13-Cys117 disulfide bridge serves as a reversible proteolytic stability switch that regulates the quantity and quality, i.e., fragmentation of the available Bet v 2. The proteolytic stability switch thus determines its immunogenicity and allergenicity.

The reduced form of Bet v 2 was found prone to dimerization and higher oligomerization states under non-reducing conditions, whereas the oxidized form of Bet v 2 remained stable as a monomer. This can be explained by the exposed cysteine 13 in the reduced Bet v 2 crystal structure. It was also reported that recombinant Bet v 2 preparation easily form oligomers under non-reducing conditions, resulting in the low IgE response in monkeys, but with substantial IgG response [22]. This oligomerization tendency of the reduced Bet v 2 form should be considered also in diagnostic tests such as the skin prick test, which could lead to false negative results.

Here we presented how a disulfide bridge can critically impact the proteolytic processing and may in turn influence the immunogenicity of Bet v 2. However, we should emphasize that our finding serves as a paradigm that is more generally applicable. For instance glycosylation, lipidation, or phosphorylation may similarly affect proteolytic processing, MHCII loading and presentation. While there are endolysosomal hydrolases such as glycosidases, lipases and phosphatases, which remove most of such modifications, these reactions are never complete and may be further regulated by pH or inhibitors as found in distinct sub-compartments of the endolysosome. Our example thus nicely illustrates that even a minority fraction of post-translationally modified proteins may have drastically different proteolytic accessibility and may thus become the immuno-dominant protein species. We therefore propose that the post-translational modifications of allergens deserve much higher attention both for sensitization and also the IgE-mediated allergic reactions.

## 4. Materials and Methods

### 4.1. Cloning, Expression and Purification of Bet v 2

Total RNA was isolated from commercial birch pollen (Ängelholm, Sweden). Pollen (0.7 g) was ground and homogenized in 10 mL extraction buffer (4 M guanidine thiocyanate, 4 mM BES pH 7.2, 4 mM EDTA, 0.01% β-mercaptoethanol, 1% *N*-lauroylsarkosine, 0.008% *n*-butanol). Supernatant was obtained by centrifugation for 5 min at 900 g, 4 °C and used for total RNA isolation using the Macherey-Nagel NucleoSpin^®^ RNA II kit (Macherey-Nagel GmbH & Co. KG, Düren, Germany) according to the manufacturer’s instructions. Reverse transcription of the Bet v 2 gene was performed using the SuperScript III RTS One-Step RT-PCR Kit (Invitrogen, Grand Island, NY, USA) according to the manufacturer’s specifications. Bet v 2 was amplified with Bet v 2 specific primer pair (Forward 5′-GAGACATATG TCGTGGCAAACGTACGTG-3′; Reverse 5′-GAGAGAATTCCTACAGGCCCTGGTCAATAA-3′) and cloned into the pHis-Parallel2 vector using the NdeI and EcoRI restriction sites. The vector containing Bet v 2 was verified by DNA sequencing and transformed into BL21 Star (DE3) expression host.

Overexpression was induced with 0.5 mM IPTG when the cell concentration reached an OD 600 nm of 0.6 at 37 °C for 4 h. Cells were harvested by centrifugation and resuspended in TBS buffer (50 mM Tris-HCl pH 8.5, 150 mM NaCl). The cells were then lysed by ultra-sonication. Upon centrifugation at 4 °C, clear cell lysate was filtered and subjected to affinity purification using poly-l-proline column. The column was prepared by coupling poly-l-proline (Sigma, St. Louis, MO, USA) onto NHS-activated agarose beads (Thermo Fisher Scientific, Waltham, MA, USA) according to manufacturer protocol. The column was first washed with two column volume of TBS buffer and further washed with five column volume using the same buffer (Figure S5). The later fraction contained relatively pure Bet v 2 which we termed it as Bet v 2 reduced form. The remaining bound Bet v 2 was then eluted with five column volume of TBS buffer containing 6 M Urea. The eluted fraction was then dialyzed against TBS buffer at 4 °C for 72 h and we termed this fraction as Bet v 2 oxidized form. Both reduced and oxidized forms of Bet v 2 were subjected to final polishing with Superdex S75 10/300 GL (GE Healthcare, Milwaukee, WI, USA) size exclusion chromatography into storage buffer (10 mM Tris-HCl pH 7.5, 20 mM NaCl). The protein concentration was determined by absorption at wavelength 280 nm and extinction coefficient of 17,085 M^−1^ cm^−1^.

### 4.2. Mass Spectrometry Analyses

For the determination of the intact mass of Bet v 2, 1 μg of protein either reduced or untreated (disulfide-bridged) protein was desalted with C18 ZipTips (EMD Millipore, Billerica, MA, USA). Using direct infusion at a flow rate of 1 μL/min, mass determination was done with a Q Exactive Orbitrap mass spectrometer (Thermo Fisher Scientific, Bremen, Germany) at maximum resolution. Background peaks of the solvent were used for lock mass calibration, resulting in an accuracy of less than 2 ppm. For deconvolution of the raw data, the Xtract modul of Xcalibur (Thermo Fisher Scientific) was used. To determine intramolecular disulfide bridges, Bet v 2 were digested with the ProteoExtract All-in-One Trypsin Digestion Kit (EMD Millipore, Billerica, MA, USA) omitting the reduction/alkylation step. After the digest, samples were desalted using C18 ZipTips. Resulting peptides were separated by reverse-phase nano-HPLC (Dionex Ultimate 3000, Thermo Fisher Scientific, column: PepSwift Monolithic Nano Column, 100 μm × 25 cm, Dionex). The column was developed with an acetonitrile gradient (Solvent A: 0.1% (*v*/*v*) FA/0.01% (*v*/*v*) TFA; solvent B: 0.1% (*v*/*v*) FA/0.01% (*v*/*v*) TFA/90% (*v*/*v*) ACN; 5–45% B in 60 min) at a flow rate of 1 μL/min at 55 °C). The HPLC was directly coupled via nano electrospray to the Q Exactive mass spectrometer (Thermo Fisher Scientific). After deconvolution with Xtract, the mass list of uncharged signals was exported to xQuest [23] and cross-linked peptides were matched to the Bet v 2 sequence with xBobcat. For sequence determination, tryptic peptides were HPLC-separated as described above and fragmented in the mass spectrometer with a top 12 method using a normalized fragmentation energy of 27%. Sequence analyses were performed with Peaks Studio 8 (Bioinformatics Solutions, Waterloo, ON, Canada).

### 4.3. Crystallization and Structure Determination

The purified oxidized Bet v 2 was concentrated to 10 mg/mL using an ultrafiltration unit with a 5 kDa cutoff membrane. To obtain a reduced form of Bet v 2, concentrated oxidized Bet v 2 was preincubated with 50 mM DTT. Hampton index screens (Hampton Research, Aliso Viejo, CA, USA) were set up to search for suitable crystallization conditions with a protein to drop ratio of 1:1 in a 96-well-plate (Art Robbins Instruments, Sunnyvale, CA, USA). Drops were equilibrated at 293 K. Bet v 2 oxidized form crystals were obtained in condition consisting of 0.1 M HEPES pH 7.5, 0.2 M magnesium chloride hexahydrate, 25% PEG3350, while the reduced form crystals were obtained in 0.1 M Tris pH 8.5, 2.0 M ammonium sulfate. The oxidized and reduced Bet v 2 crystals were cryo-protected with 35% PEG3350 and 20% ethylene glycol respectively and flash frozen in liquid nitrogen. Diffraction datasets were collected at the ESRF beamline ID30a3 and processed using XDS [24]. The structure of both Bet v 2 oxidized and reduced forms were solved by molecular replacement using existing birch pollen profilin structure (PDB:1CQA). Molecular replacement was carried out using the CCP4 software suite [25]. Structure models were built using COOT [26] and refined using Phenix [27]. Coordinates and data sets are deposited with the protein data bank (entries 5NZB and 5NZC). Since the crystal forms for the reduced and oxidized Bet v 2 differ significantly in their crystal lattice, including unit cell constants and solvent content, the two crystal lattices served to select a certain redox state, i.e., an oxidized Bet v 2 protein would be incompatible with the reduced crystal form and would, therefore, not be incorporated into the lattice, even if some oxidized Bet v 2 protein is present in solution.

### 4.4. Proteolytic Processing Assay

Human cathepsin S was overexpressed, purified and activated as described previously [20]. Cathepsin S and Bet v 2 were incubated with a molar ratio of 1 to 20 in digestion buffer (50 mM Bis-Tris pH 5.5, 100 mM NaCl) at 37 °C for 10, 20 and 30 min. At the indicated time point, samples were collected and heated at 95 °C for 5 min. All samples were then analyzed on 18% SDS-PAGE under non-reducing conditions. As a control, Bet v 2 was incubated at the same conditions without the presence of cathepsin S. Processing of Bet v 2 by human legumain was performed in the same condition by replacing cathepsin S. Human legumain was prepared as described in [28].

### 4.5. Patients and Sera

Upon case history, positive in vivo skin prick test and in vitro IgE detection (CAP system, Thermo Fisher Scientific, Phadia AB, Uppsala, Uppland, Sweden), birch pollen allergic patients reactive to Bet v 2 were selected (*n* = 7). Informed consent was obtained from all subjects included in the study and experiments with patients’ sera were approved by the ethics committee of the Medical University of Vienna (no. EK028/2006).

### 4.6. Enzyme-Linked Immunosorbent Assay (ELISA)

MaxiSorp™ flat-bottom plates (Nunc, Roskilde, Zealand, Denmark) were coated with purified oxidized and reduced forms of rBet v 2 (100 ng/well in 50 μL PBS) overnight at 4 °C. Following a blocking step, plates were incubated with 50 μL/well patients’ sera (*n* = 7) diluted 1:10 overnight at 4 °C. Detection of bound human IgE was performed with alkaline phosphatase-conjugated monoclonal anti-human IgE antibody (BD Biosciences, San Jose, CA, USA) and measured at 405/492 nm.

### 4.7. RBL Mediator Release Assay

Mediator release assays were performed according to a protocol adapted from [29]. RBL-2H3 rat basophilic leukemia cells transfected with the human IgE receptor Fcε type-1 were passively sensitized with Bet v 2-allergic patient sera by overnight incubation at 37 °C, 7% CO_2_. Every patient’s serum was previously pre-incubated 1:10 in a AG-8 cell (ATCC, Germany) suspension resuspended in tissue culture medium (MEM) with Earle´s salts (PAN-Biotech, Aidenbach, Germany) supplemented with 5% inactivated FCS (PAN-Biotech, Germany), 4 mM L-glutamine (PAN-Biotech, Germany) and the antibiotic geneticin (diluted 1:100 in medium) (Sigma, St. Louis, MO, USA) in order to deplete complement factors. After washing the cells with Tyrode’s buffer (containing 9.5 g/L Tyrode’s salts (Sigma), 1 g/L sodium bicarbonate and 0.1% (*w*/*v*) BSA), serial 100 μL/well dilutions of the antigens (each in nine dilution steps starting with 10,000 ng/mL until 0.0001 ng/mL as well as a 0 ng/mL control) in Tyrode’s D2O (Tyrode’s buffer diluted 1:1 in deuterium oxide (Sigma)) were added to the cells, resulting in an antigen-dependent β-hexosaminidase release into the supernatant. The release was measured by enzymatic cleavage of the fluorogenic substrate 4-methylumbelliferyl-*N*-acetyl-β-glucosaminide (Sigma, USA) after 1 h of incubation at 37 °C and the addition of 100 μL/well of 0.2 M glycine solution (pH 10.7) at an excitation wavelength of 360 nm and an emission of 440 nm. The release was expressed as percentage of release from cells sensitized with patient’s serum corrected for spontaneous release (just buffer, no serum) divided through total release. The total release of RBL cells was obtained by lysing the cells with 10% Triton X-100 (Amresco, Solon, OH, USA). Afterwards, curves were normalized, meaning the 0 ng/mL control value of each serum was subtracted from the measurement values. Serum obtained from a Bet v 2-non allergic individual was used as a negative control for IgE-mediated degranulation.

### 4.8. NMR One-Dimensional Spectrum

NMR spectra were recorded at 298 K on 600 MHz Avance III HD Bruker spectrometer equipped with a QXI probe and four channels for 1H/13C/15N/31P. The Bet v 2 samples at 0.2 mM protein concentrations were measured in a buffer consisting of 10 mM Tris pH 7.5 and 20 mM NaCl containing 7% D2O in 5 mm Shigemi tubes. Standard 1D 1H spectra with a WATERGATE (WATER suppression by GrAdient Tailored Excitation) scheme for solvent suppression using a 3-9-19 pulse train were recorded with 32 scans and an interscan delay of 2 s. All spectra are referenced to 4,4-dimethyl-4-silapentane-1-sulfonic acid (DSS) using the 2 mM Sucrose/0.5 mM DSS standard sample as an external reference. All spectra were processed and analyzed with Topspin 3.1 (Bruker, Billerica, MA, USA).

### 4.9. Circular Dichroism (CD) Spectroscopy

Secondary structure elements were determined using CD spectroscopy. The measurement was performed using a JASCO J-815 spectropolarimeter fitted with a PTC-423S Peltier-type single position cell holder (Jasco, Japan). Spectra were recorded ranging from a wavelength of 190 to 260 nm and presented as mean residue molar ellipticity. The samples were suspended in a 10 mM potassium phosphate buffer pH 7 at a concentration of 0.1 mg/mL. The background signal (buffer only) was recorded and subtracted from each spectrum. Secondary structure contents were calculated using CDNN CD Spectra Deconvolution software version 2.1 (Applied Photophysics, Leatherhead, UK).

### 4.10. Fourier Transform Infrared (FTIR) Spectroscopy

The proteins at a concentration of 0.5 to 1.5 mg/mL were analyzed at a constant temperature of 25 °C using an AquaSpec transmission cell adapted to a Tensor II FTIR system (Bruker Optics Inc., Billerica, MA, USA). Amide bands were recorded at a wavenumber ranging from 1500 to 700 cm^−1^. Recorded spectra were analyzed with the OPUS spectroscopy software 6.0 (Bruker Optics Inc., USA). The 2nd derivative of amide I bands was calculated by applying the Savitzky–Golay algorithm on the vector normalized spectra (25 smoothing points). The secondary structure elements were quantified via a Quant2 method of the OPUS software.

### 4.11. Thermal Shift Assay

The thermal stability of Bet v 2 was accessed through incubation with SYPRO Orange dye (Thermo Fisher Scientific, USA) and recorded using an Applied Biosystems 7500 RT-PCR thermocycler (Thermo Fisher Scientific, USA). The protein concentration was 0.3 mg/mL in assay buffer (50 mM Tris, 100 mM NaCl, pH 7.4) with or without 10 mM of DTT or 0.3 mg/mL of poly-l-proline (molecular weight from 1–10 kDa, Sigma-Aldrich, USA). The reaction was then filtered (0.2 μm centrifugation device, Pall Life Sciences, Port Washington, NY, USA) prior to mixing with a final concentration of 2× SYPRO Orange dye (excitation, 280 and 450 nm; emission, 610 nm). Each reaction was carried out in a volume of 25 μL in triplicates and heated in an Applied Biosystems 7500 RT-PCR thermocycler from 20 to 95 °C in 1 °C increments for 1 min. The results were analyzed using GraphPad Prism v5.0 (GraphPad Software, Inc., La Jolla, CA, USA).

### 4.12. Sequence Alignment and Other Computational Analysis

Multiple amino acid sequence alignment was done using CLUSTAL O (version 1.2.4) (http://www.uniprot.org/align/) multiple sequence alignment tool. Program Superpose of CCP4 suite was used to calculate Cα-rmsd.5. Statistical analyses were performed using GraphPad Prism 5 (version 5.0a).

## Figures and Tables

**Table 1:** Quantification of cross-linked (S-S) and free-cysteine (S-H) peptides in oxidized (disulfide-bridged) and reduced (no disulfide) forms of Bet v 2 by MS after tryptic digest.

Bet v 2	S-S (%)	S-H (%)
Oxidized	100	0
Reduced	11	89

**Table 2:** Crystallographic data and refinement statistics.

PDB Code	5NZB	5NZC
Structure	Oxidized Bet v 2	Reduced Bet v 2
Data collection		
Wavelength (Å)	0.9677	0.9677
Unit cell parameters		
*a*, *b*, *c* (Å)	31.76, 57.34, 59.04	74.40, 90.52, 83.17
*α*, *β*, *γ* (degrees)	*α* = *β* = *γ* = 90	*α* = *β* = *γ* = 90
Space group	P2_1_2_1_2_1_	C222_1_
Solvent content (%)	34.61	49.79
Protein chains in AU	1	2
Resolution range (Å)	41.13–1.69	41.59–2.00
Highest resolution shell (Å)	1.80–1.69	2.12–2.00
Unique reflections	12,513 (1969)	19,093 (3009)
Redundancy	16.12 (16.47)	5.67 (5.63)
CC1/2 (%)	99.8 (92.4)	99.9 (85.5)
Completeness (%)	99.6 (99.1)	98.4 (97.2)
*R_merge_	0.079 (0.640)	0.074 (0.547)
*R_meas_	0.082 (0.660)	0.081 (0.605)
Average *I*/*σ*(*I*)	20.21 (4.37)	14.35 (3.13)
Refinement		
*R*_work_ (%)	19.48	20.58
*R*_free_ (%)	22.44	23.22
Mean B value (Å^2^)	29	34
B from Wilson plot (Å^2^)	23.1	30.7
RMSD bond length (Å)	0.009	0.006
RMSD bond angles (degrees)	0.989	0.905
No. of amino acid residues	132	124
No. of water molecules	99	91
Ramachandran plot		
Most favored regions (%)	96	98
Additional allowed regions (%)	4	2

Values of highest resolution shell are given in parenthesis. *R_merge_ = Σ_h_Σ_j_ |*I*_hj_ − 〈ỗ*I*_h_〉|/Σ_h_Σ_j_|〈*I*_h_〉|; *R_meas_ = Σ_h_√(*n*/(*n* − 1)) Σ(j = 1)^*n*|*I*_hj_ − 〈*I*_h_〉|/Σ_h_Σ_j_|〈*I*_h_〉|.

## Supplementary Materials

Supplementary materials can be found at www.mdpi.com/1422-0067/18/10/2156/s1.

## Abbreviations

DTT	Dithiothreitol
MHC	Major histocompatibility complex
PDB	Protein data bank
RBL	Rat basophil leukemia
IgE	Immunoglobulin E
MS	Mass spectrometer
NMR	Nuclear magnetic resonance spectroscopy
CD	Circular dichroism spectroscopy
FTIR	Fourier transform infrared spectroscopy
RMSD	Root-mean-square deviation
ELISA	Enzyme-linked immunosorbent assay
IPTG	Isopropyl β-d-1-thiogalactopyranoside
TBS	Tris-buffered saline
TFA	Trifluoroacetic acid
ACN	Acetonitrile
ESRF	European synchrotron radiation facility
ATCC	American type culture collection
FCS	Fetal calf serum
BSA	Bovine serum albumin
EDTA	Ethylenediaminetetraacetic acid
BES	*N*,*N*-bis(2-hydroxyethyl)-2-aminoethanesulfonic acid
