# Supplementary material for: Two Distinct Conformations in Bet v 2 Determine Its Proteolytic Resistance to Cathepsin S
Source: Int J Mol Sci. 2017 Oct 16;18(10):2156. doi: 10.3390/ijms18102156 (PMC5666837; doi:10.3390/ijms18102156)
Supplement: Supplementary file 1 [file ijms-18-02156-s001.pdf]

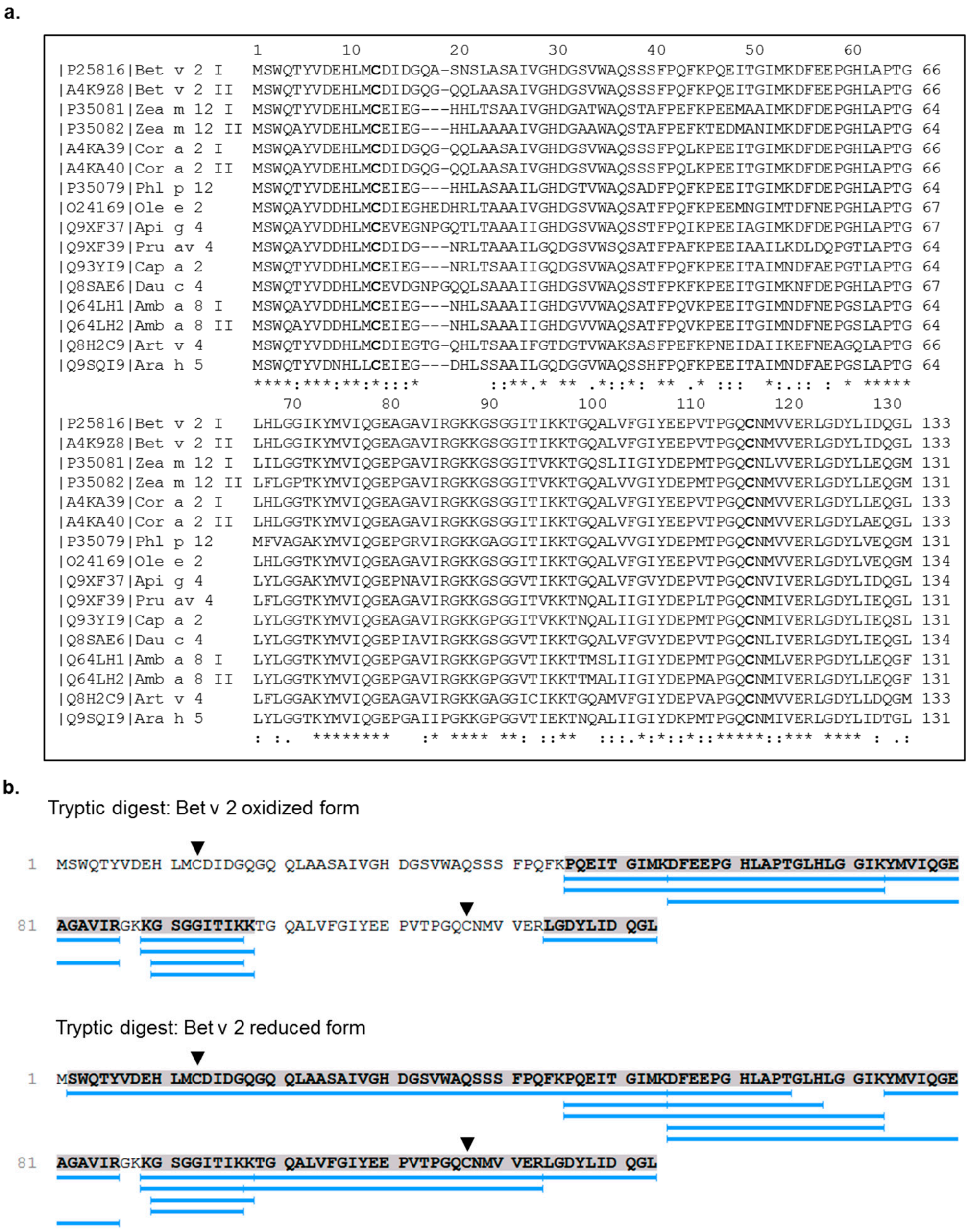


**Figure S1.** Identity of Bet v 2. (a) Multiple amino acid sequence alignment of profilin allergens. Panel left indicated the uniprot entries codes. Two conserved cysteine residues were indicated by arrows. (b) Bet v 2 tryptic digest peptide mapping sequence coverage by Mass Spectrometry. (b) Peptide mapping of Bet v 2 by mass spectrometry and *de novo* sequencing with PEAKS. Both forms of Bet v 2 were digested with trypsin without prior reduction/alkylation. Cys-containing peptides were not detected in the oxidized form. This indicates that all its cysteine residues were involved in disulfide bridges, since the software algorithm of PEAKS is only able to identify linear peptides. The cross-link between Cys13 and Cys117 was verified using xQuest (see Table 1). Cys13 and 117 were indicated with arrows.

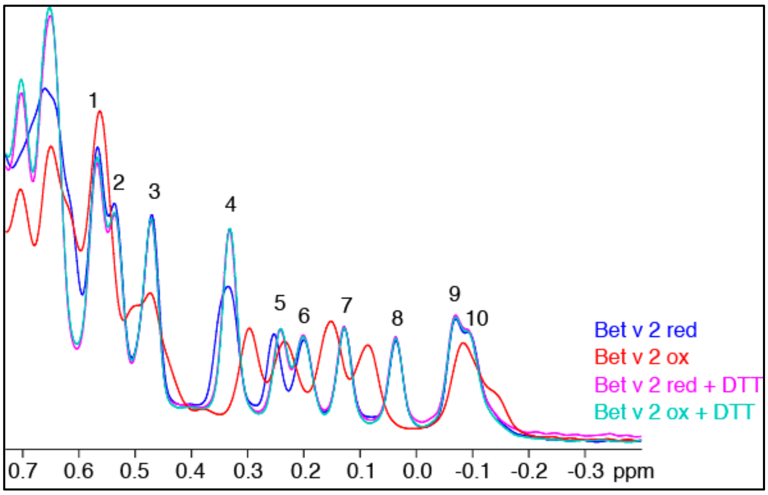


**Figure S2.** One-dimensional $^{1}H$ spectra of 0.2 mM Bet v 2 preparations in Tris buffer measured at 298 K and 600 MHz using 32 transients.

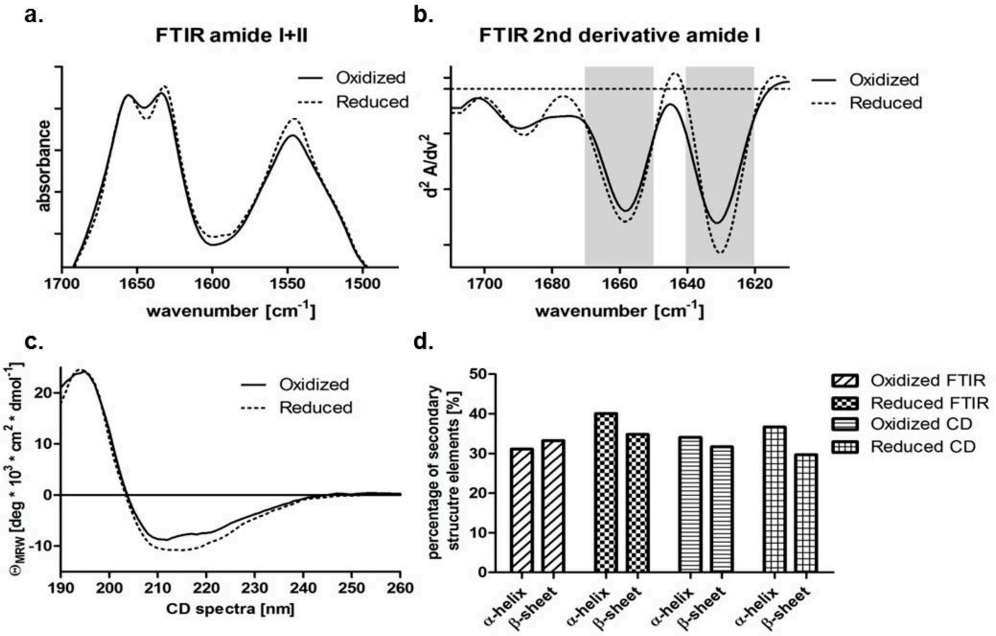


**Figure S3.** Secondary structure content of Bet v 2 oxidized and reduced forms. (a) FTIR amide I and II spectra. (b) FTIR second derivative of amide I spectra. (c) Circular Dichroism spectra. d. Summary of calculated alpha helixes and beta sheets content for FTIR and CD.

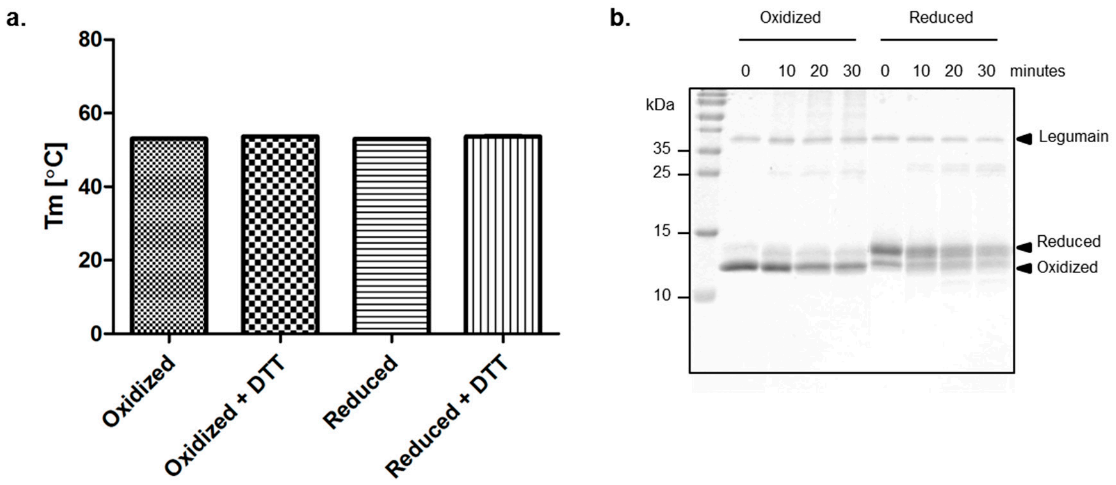


**Figure S4.** Stabilities of Bet v 2. (a) Thermal stability of Bet v 2 determined by thermal shift assay. The assay was performed in triplicates and the error bar was too small to be seen (b) Proteolytic susceptibility of Bet v 2 towards Legumain. Chronological digestion assay was performed at pH 5.5, 37°C up to 30 minutes with legumain to Bet v 2 molar ratio of 1:20. Digestion profiles were visualized on SDS-PAGE under non-reducing condition and Coomassie Blue staining.

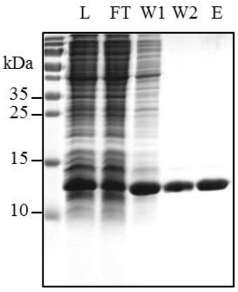


**Figure S5.** Purification of recombinant Bet v 2. Proteins were visualized on a reducing SDS-PAGE and stained with Coomassie Blue. L, cell lysate; FT, flow through; W1, wash (2 column volume); W2, wash (5 column volume); E, elution.
